# Recent Advances in Sensors for Fire Detection

**DOI:** 10.3390/s22093310

**Published:** 2022-04-26

**Authors:** Fawad Khan, Zhiguang Xu, Junling Sun, Fazal Maula Khan, Adnan Ahmed, Yan Zhao

**Affiliations:** 1 College of Textile and Clothing Engineering, Soochow University, Suzhou 215123, China; 20187215005@stu.suda.edu.cn (F.K.); 20187215006@stu.suda.edu.cn (A.A.); 2 China-Australia Institute for Advanced Materials and Manufacturing, Jiaxing University, Jiaxing 314001, China; 3 Shandong Qingdao Petroleum Branch, SINOPEC Sales Co., Ltd., Qingdao 266071, China; sunjunling0935@dingtalk.com; 4 School of Materials Science and Engineering, Beihang University, Beijing 100191, China; fazal@buaa.edu.cn

**Keywords:** fire detection, sensor, heat, flame, gas, smoke

## Abstract

Fire is indeed one of the major contributing factors to fatalities, property damage, and economic disruption. A large number of fire incidents across the world cause devastation beyond measure and description every year. To minimalize their impacts, the implementation of innovative and effective fire early warning technologies is essential. Despite the fact that research publications on fire detection technology have addressed the issue to some extent, fire detection technology still confronts hurdles in decreasing false alerts, improving sensitivity and dynamic responsibility, and providing protection for costly and complicated installations. In this review, we aim to provide a comprehensive analysis of the current futuristic practices in the context of fire detection and monitoring strategies, with an emphasis on the methods of detecting fire through the continuous monitoring of variables, such as temperature, flame, gaseous content, and smoke, along with their respective benefits and drawbacks, measuring standards, and parameter measurement spans. Current research directions and challenges related to the technology of fire detection and future perspectives on fabricating advanced fire sensors are also provided. We hope such a review can provide inspiration for fire sensor research dedicated to the development of advanced fire detection techniques.

## 1. Introduction

Fire has been a valuable gadget throughout mankind’s history, however, it can likewise bring disaster if not carefully controlled. With the advances in electronic devices, sensors, information communications, and technologies, the construction industry is experiencing a transformation. This has led to the emergence of many technological developments. The digital revolution has considerably aided in cutting running expenses while also improving performance. Likewise, when materials and insulation technologies improve and become more widely used in building constructions, the risk of loss of life and financial assets as a result of fire increases. Fire vulnerability is an unceasing danger in daily life. Ever since the late 1900s, there has been a considerable drop in the number of fire deaths due to increased usage of technologies to prevent or stop fires, such as smoke detectors, sprinklers, and emergency evacuation plans. Even with all these advancements, fire remains a significant concern, costing roughly 1% of global GDP each year and resulting in the loss of thousands of lives. Recent fire tragedies include the Lebanon Beirut explosion in 2020, the 2019–2020 Australian bushfires, the Notre Dame de Paris (Gothic cathedral) fire in 2019, the London Grenfell Tower fire in 2017, etc. From 2013 to 2017, the “International Association of Fire and Rescue Services” reported an average of 1097 deaths per year (~4 deaths/day) in fire accidents around the world [1]. This has led in recent years to the emergence of several new techniques for fire detection and prevention. Therefore, a greater emphasis is put on the construction of smart buildings and structures worldwide [2].

Over the last decade, several novel fire detection technologies have been created with advancements in sensors, IT and microelectronics, as well as the in-depth understanding of fire physics. Techniques for measuring practically every stable gaseous species generated before or during combustion are now available. The introduction of distributed optical fiber temperature sensors in applications with difficult climatic conditions, such as tunnels, underground railways and stations, can provide fire prevention [3]. Various fire elements, such as smoke, heat, and carbon monoxide, are detected by multiple sensors, and a complicated algorithm is used to intelligently discern the difference between fire and non-threatening conditions. Furthermore, fire alarm systems are combined with other building facility systems to eliminate false alarms, accelerate the evacuation of buildings and aid in firefighting [4]. According to the National Fire Protection Association (NFPA), in the United States, the number of major “house” fires has dropped down in recent years, partly because fire detectors have been introduced into residential buildings [5]. On the other hand, however, in the last decade, natural materials such as wood have been replaced by synthetic materials in thermal insulation, structural materials, furniture, and finishes. As a result, the risk to life and property has shifted dramatically, because the combustion of synthetic materials not only produces very harmful poisonous smoke but also releases much more carbon dioxide than natural materials [6], resulting in a shorter escape time. Many of the places most in need of protection are unattended, such as telecommunication facilities, and the service interruption caused by fire becomes more and more expensive. In certain situations, a fire can only be found after it has fully developed, which will seriously damage property or cause loss of life. To better safeguard the public and fulfill evolving requirements, fire detection technology still confronts hurdles in decreasing false alerts, improving sensitivity and dynamic responsibility, and providing protection for costly and complicated installations.

In recent years, the development in fire sensors has been reviewed and summarized from several perspectives: chemical sensors associated with fire detection [7], fire detection algorithms [8], video fire detection [9], video smoke detection [10], sensors modules [11], fire monitoring systems [12], forest fire detection [13], distributed heat sensors [14], and fire sensors for specific location [15] and extreme conditions [16]. However, none of them provides a comprehensive analysis that covers all of the highlighted and emerging fire detection technologies to date, as well as the discussion of what further improvements can be made. The purpose of this paper is to review recent fire detection technology research and development, including emerging sensor technology, fire signal processing and monitoring technology, and an integrated very early fire detection system for building fires. Some concerns and potential operations related to the technology of fire detection are discussed and compared, and future directions and perspectives on fabricating advanced fire sensors are also provided.

## 2. Stages of Fire and Structural Designs for Fire Risk Mitigation

Ignition, fire growth, and eventually flashover, followed by a cooling phase, are the stages of fire development in enclosures. The primary concern throughout the ignition and growth phases is lifesaving, which is where fire sensors play a big role by warning and alarming. The first 10 to 15 min of a building fire, during which the residents may leave for safety, are the most critical in terms of life safety. The fire is described as a pre-flashover fire because it is restricted by the amount of available pyrolyzed gaseous fuel. When a flashover happens, the fire becomes fully developed. Post-flashover fire is described as fire that burnt at its maximum capability with the available air supply [17].

Flashover is a highly dangerous and life-threatening situation for firefighters. The time it takes for a room to reach this level varies, depending on the room’s size and geometry, combustible contents, air supply, room insulation, and the chemistry of the hot top layer [18]. Studies have been conducted to calculate the temperature [19], predict its occurrence [20,21], minimize its effect [22], and construct a design that can withstand the temperature of post-flashover [23]. A flame will enter the decay phase when it reaches the decline stage. After the fully grown flame runs out of fuel or oxygen, this stage occurs. By limiting oxygen supply using firefighting equipment, fires can be driven into the decay stage.

Post-earthquake fires (PEFs) are a damaging hazard induced by big earthquakes that can result in significant financial and human losses [24]. Large earthquakes can destroy structural and non-structural components, including fire extinguishing systems, fallen ceilings, and partitions, all of which can exacerbate the formation of PEFs [25]. When fire extinguishing systems are damaged, their ability to extinguish fires is dramatically reduced, causing smoke and fire to spread throughout the building and jeopardizing a safe evacuation process. It is critical to use building information modeling (BIM) as part of the modeling and planning for the rescue procedure [26]. Lu et al. [27] proposed a simulation framework based on BIM and virtual reality (VR) for an indoor post-earthquake fire rescue scenario, which includes a smoke visualization method that combines volume rendering and the particle system. The findings suggest that the developed VR scenario has a high level of realism and flawless interactivity, and that smoke has a greater impact on fire rescue than fallen debris. Lofti et al. [28] provided a feasible and practical model for evacuation assessment during a PEF using BIM and PyroSim, which included simulated smoke and fire developments in various floors, implying that additional equipment, such as smoke curtains and rescue chute should be provided in different floors.

## 3. Fire Sensors

**Figure 1:**
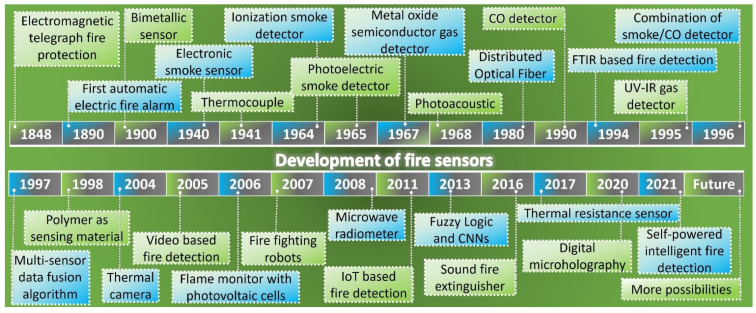
A brief timeline on the development of fire sensors.

**Figure 2:**
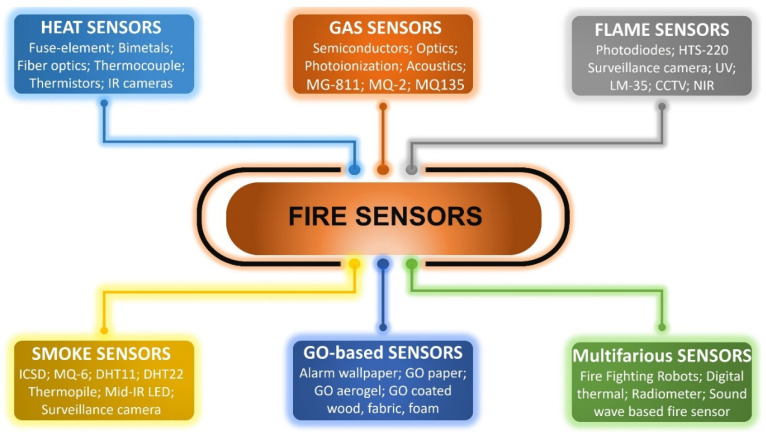
A summary of current fire sensing technologies.

Technology for fire detecting depends on the fire’s location and nature. In this review, the application and implementation status of alarm systems, advantages of the latest technologies, as well as their limitations and shortcomings are explained. Over a period of time, numerous fire detecting technologies have evolved. Some of these methods are still in use today, while others are becoming obsolete. A brief timeline of the development of fire sensors is given in Figure 1. The key five detecting methods, comprising heat, gas, flame, smoke, and graphene oxide (GO) based sensing, as well as other fire sensing technologies and current research, are explained in depth. In fire detection based on heat sensing, most current fire detection systems use electronic and distributed optical thermal detectors based on thermistors. Thermal sensing based on infrared is a helpful technique, especially appropriate for thermal detection of the targeted location. Compared with non-visual methods, visual approaches for smoke and flame detecting are attracting research focus because of their rapid response time and low imprecision output rate. The great algorithmic capability of existing digital systems has opened up new research prospects for deep learning of complicated neural network-based technologies. Among different gas sensing technologies, gas sensors based on semiconductor metal oxides have been useful in practice due to their great sensibility, small size and reduced cost. However, they have problems with stability, which require more study to resolve. Currently, research is also underway in the field of carbon nanotube-based gas sensing for fire detection. Microwave radiometer-based fire sensing is one of the most important contemporary approaches due to its key benefit of fire detection across barriers such as walls. For fire detection, the multi-sensor fusion method based on wireless sensor networks (WSNs) and the Internet of Things (IoT) is suitable. The new emerging technique of graphene oxide (GO) based sensing has shown an outstanding short response time. Given the combustible materials, the property of fire-resistant and fire sensing can certainly reduce fire incidents, but further work still has to be done for its practical implementation. These current fire sensing techniques are illustrated in Figure 2.

### 3.1. Heat Sensors

**Figure 3:**
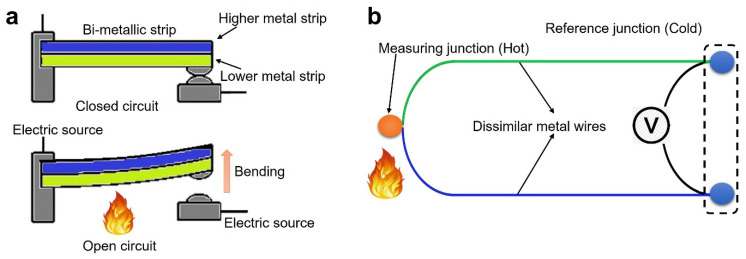
Heat sensors: (**a**) bi-metal strip sensor working principal; (**b**) thermocouple working principal.

Heat sensors are used to measure the ambient heat in a residence because of the occurrence of fires. The sensors are sensitive to the temperature that is related to the resistance variation, displacement, and refractive index, etc. [29]. Generally, there are three types of heat sensors: fixed temperature, increase rate, and compensation rate. The fixed temperature heat sensor is triggered when the temperature rises above the threshold value, which is at or above 60 °C. There are many categories of fixed temperature sensors, such as fuse-element, bimetals, and distributed fiber optics. The fuse-element type heat sensor operates at a set temperature level based on the melting of the heating element and is mainly used in the fire sprinkler system. The bi-metal heat sensor works according to the mechanism of thermal expansion of the metals (Figure 3a). When the temperature rises, the bimetallic strip will bend to the metal with a low coefficient of thermal expansion. Distributed heat sensors are further divided into three groups: electrical, sheathed thermocouples and optical. The electrical and sheathed thermocouple heat sensors work according to the principle of change in the wire resistance and surface temperature (Figure 3b) [30]. Electronic type heat detectors operate with a thermoelectric effect, which uses one or two thermistors to detect the temperature [31]. The type of optical distributed heat sensor which finds its application in mines, tunnels, and underground pipelines is operated by the back-scattered light pattern principle [32]. The heat sensor is built with a minimal working temperature or a threshold temperature environment. The rate of compensation heat sensor is enabled when the air temperature is higher than the fixed temperature. Further discussion on each type of heat sensor is given as follows.

#### 3.1.1. Distributed Optical Fiber Heat Detectors

One of the most favorable heat monitoring technologies for fire safety applications is the distributed optical fiber temperature sensor [33]. The optical fiber sensor, unlike ordinary heat sensors, uses the entire optical fiber as the detecting medium. Temperature measurements can be carried out along the fiber optic cable at any and every point. The temperature that is recorded ranges from 160 to 800 °C, and is only restricted by the fiber’s durability or, more accurately, by its main coating. The optical fiber sensor cable reacts rapidly to temperature variations owing to its low mass compared to traditional heat sensors. The fiber cable itself is sturdy, resilient, and adaptable to a variety of geometries, and could be installed directly in or near protected areas. They have been utilized to provide fire protection in some applications with challenging environmental circumstances, such as tunnels, conveyor lines, subterranean railways and stations, steelworks, and petrochemical industries [34].

**Figure 4:**
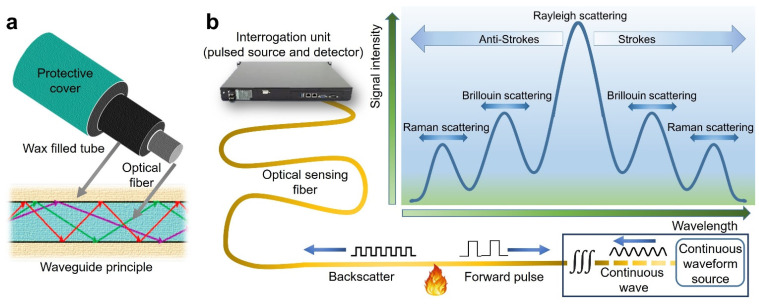
Optical fiber: (**a**) typical cross-section and waveguide principle; (**b**) basic scheme of fire sensing setup and different scattering components in optical glass fibers.

Distributed optical fiber heat sensors were implemented for fire detection based on Rayleigh or Raman scattering [35,36]. For instance, Meacham et al. [37] has invented a Rayleigh scattering cable sensor that consists of three main components: optical fiber, a wax-filled tube, and a protective cover (Figure 4a). The wax starts to melt and expand when heated at any point, creating a difference in the reflected light. The system’s maximum sensing range is up to 2 km. The system’s main disadvantage is that it does not guarantee any temperature rise over time and only has one alarm that drives temperatures between 40 and 90 °C.

The Raman scattering optical fiber sensor senses the temperature by calculating the ratio of stokes and anti-stokes backscattering intensity signals as a function of temperature. The Raman system has a maximum sensing range of up to 4 km and its spatial resolution ranges from 8 to 1 m, depending on the response time and temperature resolution requirements. The Brillouin light scattering (BLS) fiber optics system is a viable alternative to Rayleigh and Raman scattering for temperature measurement [38]. The BLS perceives the temperature change by evaluating the Brillouin backscattering intensity and its frequency variation as a function of temperature [39]. Figure 4b illustrates the general scheme of fire sensing setup and scattering components for Rayleigh, Brillouin, and Raman scattering in optical glass fibers.

In addition, Dong et al. [40] developed a low-cost, high-efficiency optical cable with a temperature sensing ability utilizing fiber Bragg grating (FBG), which blocks specific wavelengths, based on a linear relationship between FBG’s reflected wavelength and cable temperature. Wang et al. [41] also implemented an FBG array for small fire detection, which can detect small targets with excellent accuracy that are smaller than the spatial resolution. Hoff et al. [32] created a fiber optic distributed temperature sensing (DTS) based fire detection system for industrial conveyor belt fires, in addition to acoustic fire measurement. For compartment fires, Zhu et al. [42] proposed an instrumentation technique that used distributed fiber optic sensors to assess temperature through composite floor beams.

#### 3.1.2. Thermal Resistance Sensors

**Figure 5:**
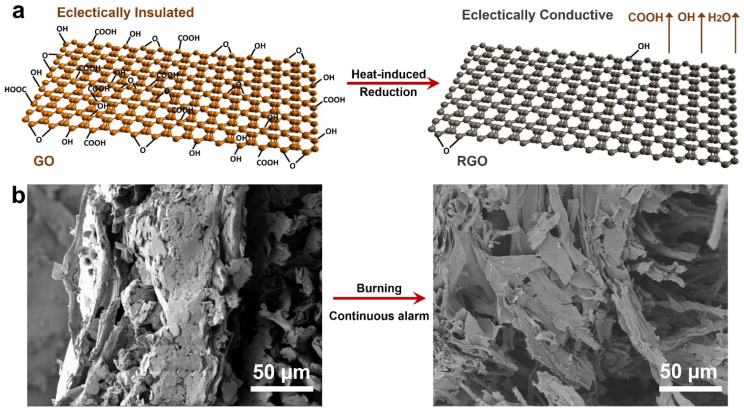
(**a**) Thermal-induced reduction of GO; (**b**) cross-section SEM images of GO/PDMAEMA/BN fabric before and after burning (Adapted with permission from Ref. [44]. 2021, John Wiley and Sons).

Based on the reduction of graphene oxide (GO) at high temperature, GO-based fire sensor has been a new approach for effective and timely detection of fire danger. Typically, GO has low electrical conductivity. When encountering fire or high temperature, GO reduces thermally to graphene (rGO) with a high electrical conductivity through the decomposition of the oxygen-containing functional groups, such as carboxyl groups, epoxy groups, and hydroxyl groups [43]. The explanation for this response behavior for detecting a flame and heat lies in the change in the electrical resistance of the GO (insulator), which turns into rGO (conductor) during the flame attacking process (Figure 5a). Another key feature of these sensors is that they should be flame resistant to ensure that the alarm signals endure as long as possible. Combustible materials (e.g., cotton, paper, wood, and foam) are used as the substrate in these sensors since they are sensitive to fire and heat and degrade quickly. As shown in Figure 5b, the cotton fabric that was employed as a substrate still maintained its structural integrity for continuous alarm after fire exposure.

As from the previous work in the field of flame retardancy and fire warning sensors, fabricating a flame retardant GO-based coating is considered to be a promising way to overcome the danger of fire hazards. For instance, Wu et al. [45] used the dip-coating method to fabricate hierarchical GO/silicone on combustible materials. On fire encounters, silicone offers the necessary flame retardancy while GO nanosheets undergo thermal reduction and provide a conduction path for the warning circuit in 2–3 s. Functional cellulose and GO were fabricated on wood, polyurethane, and polypropylene foam using the self-assembly dip-coating method by Xie et al. [46]. Another research reported by Xie et al. [47] showed an ultra-sensitive signal time that is 0.83 s, by fabricating a coating containing GO, silver nanowire (AgNW) and fluoride polyvinyl butyral (FPVB) using the spray-coating method. Xu et al. [48] fabricated melamine-formaldehyde sponge coated with GO wide-ribbon using the facile dip-coating method. The coating has good hydrophobicity, structural stability, and reversible compressibility, and can complete the alarm circuit in about 2 s. Huang et al. [49] reported silane-GO papers that display outstanding flame resistance, acidic/alkaline tolerance and mechanical flexibility, having a rapid flame detecting response time of about 1.6 s. Qu et al. [50] designed a composite film using black phosphorene-MoS_2_ nano-filler and GO that can detect fire in 1 s, and also developed a highly flexible film from black phosphorene and GO that can trigger the fire alarm in less than 1 s [51]. 

**Figure 6:**
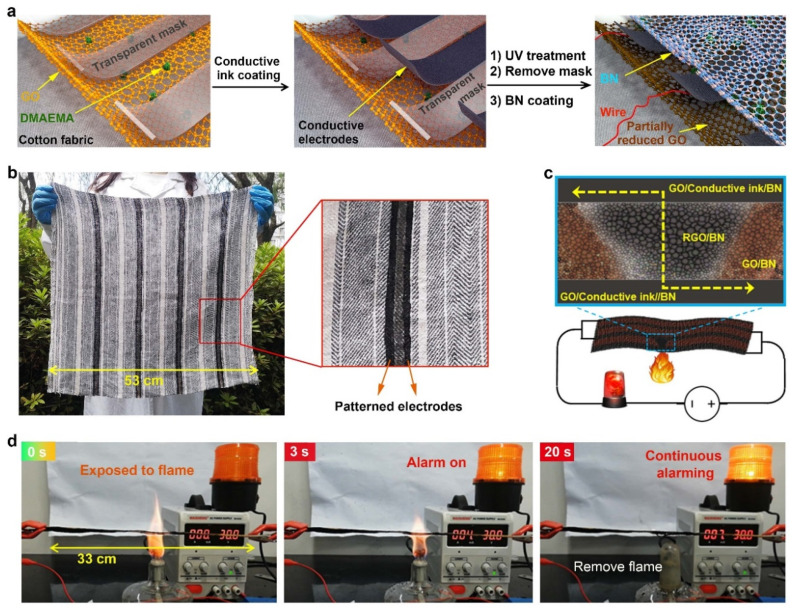
(**a**) Schematic illustration of the fabrication procedure of GO/PDMAEMA/BN and conductive ink/BN patterned fabric. (**b**) Example of a large piece of striped fabric coated with conductive electrode and fire-sensing lines. (**c**) Construction of a large-area early warning sensor by connecting the conductive ink/BN lines in an interdigitated way. (**d**) A strip of 33 cm long patterned fabric was used for the demonstration of large-area sensing. (Adapted with permission from Ref. [44]. 2021, John Wiley and Sons).

Most of the GO-based fire sensors have small detection areas. To overcome this limitation, we recently developed a large-scale sensor (>33 cm and extendable) that exhibits a short alarming time of <3 s in response to external abnormal high temperature, heat, or fire, through the use of parallel lines of conductive ink as built-in electrodes, which are coated on cotton fabric that has a hierarchical flame retardant nanocoating composed of GO, poly(dimethylaminoethyl methacrylate) (PDMAEMA) and hexagonal boron nitride (BN) (Figure 6) [44]. At any place where it encounters fire or heat, at that place, the reduction of GO takes place and will complete the circuit and thus give the alarm of fire danger. The hierarchical nanocoating can self-extinguish, thus enabling the substrate not to be burnt out, and gives a continuous long-lasting alarm. It is suitable for use indoors and outdoors, flexible if the substrate is flexible, suitable for a large area of detection, and also provides effective fire resistance to combustible materials that will certainly reduce the occurrence of fire incidence.

#### 3.1.3. Miscellaneous Heat Detectors

This section describes some of the multiplicity and, in some cases, lesser-known heat detection approaches that have been used for fire detection. For instance, a system that is able to track the temperature difference between inside and outside surfaces of the wall was proposed by Chiang et al. [52]. For the control of fire phases, the temperature change between the wall surfaces is monitored. By conducting experiments and accompanying simulations, Jevtic et al. [53] examined three distinct electric wires for fire sensing and location. Several variables of central wire (e.g., capacitance, impedance, resistance, impedance, etc.) will change with temperature. They tested a coaxial cable up to 100 m. A fire location model based on far-field and near-field was offered by Wang et al. [54], and the temperature sensor array was used to measure the fire stages. For the best location of the fire sensor, the proposed model is acceptable. However, for the capacity of fire phases, the suggested model is not sufficient. Bosch et al. [55] carried out fire detection with an infrared thermal imager using spatiotemporal features, and the flame zone is separated by the image threshold using histogram. The infrared method is ideal for fires that have low radiance due to alcohol and hydrogen. Table 1 summarizes the recent developments in the field of heat sensors.

### 3.2. Gas Sensors

Gases are emitted at every stage of combustion, and unique gas characteristics can be used to reliably detect fires. Jackson et al. [72] identified the density of CO, CO_2_, H_2_, O_2_, and smoke produced by wood fire, cotton fire, plastic fire, liquid *n*-heptane and spirit fires. The chemical composition of smoke from various types of fires varies radically, according to their source. CO is the best of the four warning gases, appearing in all six types of fires. CO fire sensors that work at room temperature, require a low-power source in comparison to traditional detectors and can protect against smoldering fire, including the combustion of organic materials in which substantial amounts of carbon dioxide are emitted early in the combustion process [73].

**Figure 7:**
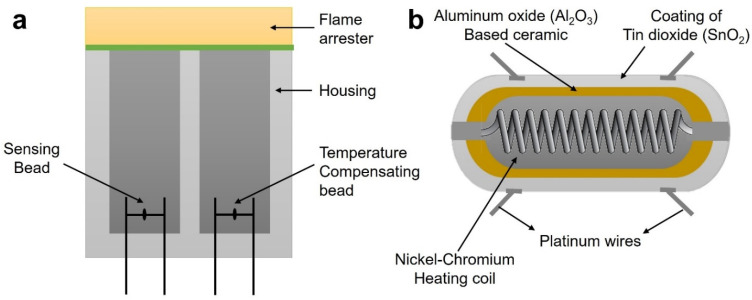
Gas sensors: (**a**) catalytic beads combustible gas sensor; (**b**) metal oxide semiconductor (MOS)-based resistive sensor.

Liu et al. [74] presented a thorough analysis of gas sensing technology and compared the selectivity and sensitivity of various gas sensing technologies. Energy consumption, response time, reversibility, adsorption capability, stability, manufacturing costs and other parameters were discussed. By measuring the change in gas sensor output, the existence of gases in a certain position is sensed by the gas sensor. The existing gas sensors are based on catalytic beads, semiconductors, optics, photoionization, acoustics, IR, electrochemistry, gas chromatography, calorimetric systems, etc. Compared with the components of good air quality, air quality standards are disturbed by fire hazards. In the case of fire hazards, a significant amount of CO_2_ is emitted [75], and the most harmful gases in a fire are CO and HCN [76]. The oxygen content drops as the CO content rises. Low oxygen concentration changes are a sign of smoldering, while a high oxygen concentration change is a warning of the combustion of liquid fuel. Catalytic bead sensors, also known as pellistors, are the most common sensing elements for detecting combustible gas concentrations in the air (Figure 7a). They can be used in mobile and stationary equipment in mines and other industries to indicate the presence of explosive conditions [77]. One of its issues is that their output signals fluctuate with changes in ambient temperature. In the event of explosive situations, it can result in either false alarms or a lack of response [78]. Numerous types of gas sensors have been used, but semiconducting metal oxide gas sensors have received a lot of attention because of their low cost, ease of operation, high stability, and ability to respond to a wide range of chemicals.

#### 3.2.1. Metal Oxide Semiconductor Gas Sensors

Metal oxide semiconductor (MOS) gas sensors have high sensitivity and a low price. The gas sensing mechanism of MOS-based sensors is primarily based on changes in resistance caused by chemical interactions between target gas molecules and adsorbed oxygen ions on the MOS surface when they are exposed to the target gases. They do, however, have stability concerns that lead to a false alarm. By applying additional layers (such as zeolites) over metal oxides, the discrimination of these sensors can be enhanced [79]. Some composite materials also demonstrate better-quality performance for gas sensors of this type [80]. A sensor array is chosen for multi-parameter gas sensors [81]. The usage of polymers in gas sensing applications has been demonstrated in the enhancement of sensitivity, because its doping levels are easily modified by chemical interactions with various analytes at ambient temperature, giving a straightforward technique for detecting gas analytes [82]. In order to detect electronic fires, Riches et al. [83] tested the sensitivity of surface acoustic wave (SAW) and metal oxide semiconductor (MOS) sensors. The SAW detector detects fire by evaluating the frequency change caused by the absorption of gas or vapor on the surface of piezoelectric crystal. The conductivity of a metal oxide sheet in organic vapor is measured by the MOS sensor, which detects fire. A micro-machined tin oxide system was developed and manufactured by Mandayo et al. [84] for CO detection. An Au/MO (metal oxide)/n-low temperature polysilicon MOS Schottky diode was designed by Juang et al. [85] on a glass substrate. Because of having a large band gap along with a high surface to volume ratio, SnO_2_ has the highest relative sensitivity ratio of multiple metal oxides, such as SnO_2_, TiO_2_, and ZnO. To detect fire-related gases, such as benzene, CO, and isopropanol, a gas sensor was developed by Abid et al. [86] using SnO_2_ nanowires. When heated to 200 °C, it detects cotton, beech, and printed circuit board smells. It operates on the principle of calculating the variation in the resistance of the components of the sub-sensor caused by the above gases. A new class of metal-oxide sensor for fire detection uses metallic heaters, such as Pt, Pd, Ag, Ni, and Cr to raise the sensor temperature to the desired value [87]. As a result, simple and inexpensive gas sensors based on device resistance measurements have been developed (Figure 7b). 

#### 3.2.2. Optical Gas Sensors

The optical gas detection method based on the principles of spectroscopy is more reliable and sensitive and has a short response time. However, with these detection approaches, the main issues are their high cost and large size. Heidari et al. [88] suggested a technique in miniaturized form to build these gas sensors. Their technology uses microfluidic penetration and is ideal for detecting CO_2_, CO, and some other gases. Dankner et al. [89] designed an electronic-optical gas sensor. It can monitor and transmit alarm signals before fire or explosion, and detect flammable paraffin, aromatics, and hydrogen sulfide at low concentrations. Furthermore, the developed gas sensor is able to work in diverse environmental conditions, including rain, fog, aqueous vapors, and nebulized gases.

#### 3.2.3. Acoustic Gas Sensors

A gas detecting method based on acoustics is reported in the literature, which displays a change in acoustic wave velocity due to the variation of particular parameters (such as mass) of sensing material [90,91]. The measuring gas is passed through by a wavelength or intensity-modulated LASER beam. The molecules of the LASER beam absorb and release energy and produce an acoustic wave that is sensed by an acoustic sensor [92]. The acoustic wave’s size offers information on gas concentration.

#### 3.2.4. Miscellaneous Gas Sensors

A classifier-based gas identification method was proposed by Shi et al. [93] and various gas identification algorithms were used to accurately detect fires. The measurement of parameters, including temperature, CO and CO_2_ can be used not just to identify early fire but also for building comfort. A wireless gas measurement device such as CO_2_ and CO with ambient temperature and humidity was developed by Kumar et al. [94]. Researchers have also investigated the use of the FTIR spectrometer for fire detection [95]. FTIR observes the spectrum from 2.5 to 25 mm and measures the existence of several species of interest, allowing for timely fire detection with minute false alarms. FTIR measurements also provide a large amount of other data before ignition and early fire, comprising monomer species, olefins, unburned fuels, oxalates, and pyrolysis products [96]. The integrated CO and smoke sensor will alleviate the inadequacies of either CO or smoke sensors in fire detection and deliver improved fire sensing by differentiating numerous nuisance sources and enhancing sensitivity [97]. Qiu et al. [98] developed an early warning fire detection system based on the wavelength modulation spectroscopic CO sensor using a 32-bit system-on-chip that can detect fire in 24 s with no false alarm.

### 3.3. Flame Sensors

The fire itself is a radiation source, which can be sensed by identifying the radiation generated in the combustion zone [99]. Flame is the visible part of fire, which is caused by the exothermic reaction between fuel and oxidant [100]. The flame temperature depends on the material being burned. It has both features of flame that are color (chromatic properties) and radiation. Centered on non-visual and visual techniques, there are two methods of flame detection. The non-visual technology is based on flame radiation, while the visual technology is based on the color of the flame. 

#### 3.3.1. Non-Visual Flame Detection

The radiation emitted by the flame depends on the temperature of the flame and the type of fuel burned. The ultraviolet, visible, and infrared sensors are available for flame sensing and categorized on the basis of their spectrum. Xu et al. [101] designed a flame sensor based on three photovoltaic cells. The three photocells test the spectral bands of IR, visible, and UV, respectively. Owing to the deposition of aerosols on receptor glass, the ratio of false positives is increased in the UV flame sensors. The UV sensors emit UV spectrum sparks that serve as a warning to interrupt the sensor. Pauchard et al. [102] suggested a model with the aid of a UV flame sensor to remove the impact of sparks. A low-cost near-infrared (NIR) photodetector for flame detecting has been described by Lacovo et al. [103] based on colloidal quantum dot (CQD) technology using a PbS semiconductor. Two SiC photodiodes were used and the relationship between the temperature variation and the shape variation of the 260–350 nm OH band was utilized for flame detection.

#### 3.3.2. Visual Flame Detection

The issue with customary fire, smoke, flame, and gas sensing sensors is transport delay. To trigger them, it takes time for particles to hit the point sensors, and thus limited coverage area is another problem. Therefore, large numbers of point sensors are needed to cover large areas. Fire has several characteristics, such as size, position, color, growth, burning degree and dynamic texture [104]. Although all of these data cannot be captured by conventional sensors, human presence is required in order to verify the rationality of these alarm signals because these traditional sensors may send out erroneous alert signals. All of these concerns can be greatly decreased by employing cameras to gather fire photos and evaluating them for fire detection. In order to minimize the cost, a surveillance camera can be used instead of a dedicated fire detection camera. The two types of cameras utilized for flame detecting are IR and visible cameras.

The flame was detected by an infrared thermal imager, and a hidden Markov model was used for flame flicker detection by Toreyin et al. [105]. The camera offers various formats of image signals, such as JPEG, RGB, RAW, etc. The algorithm is then used to process these signals to forecast fire or non-fire frames. Broadly speaking, there are two ways to design algorithms. The first method is based on learning, and it involves extracting a dataset of fire and non-fire test photos to refine the device. These are neural network-focused deep-convolution algorithms. In this direction, research is still in its early stages. In the second method, color, form, flicker frequency, and the dynamic structure of fire are significant elements. In fire detection applications, the RGB, Hue, L*a*b*, YUV, and HSI color spaces are utilized. 

**Figure 8:**
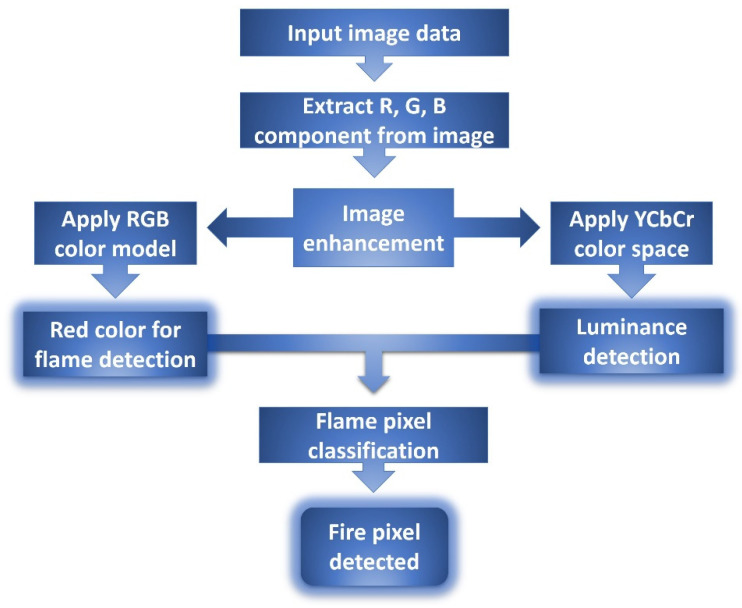
Flow chart for fire pixel detection using RGB and YCbCr color models.

For flame detection, Celik et al. [106] used YCbCr color space. This color space efficiently distinguishes brightness from chrominance. Kozeki et al. [107] explored the use of a thermal camera system to detect and monitor burning fires using appropriate image processing algorithms. The experimental results show that the effect of image processing software on the combustion stage of silk/cotton mat and that of non-fire dangers (e.g., electric radiant heater) is correct. Khalil et al. [108] proposed a model which used multicolor space and background modeling that has an average detection rate of 97.1%. Chen et al. [109] demonstrated different features of fire detection in the fusion method, that is flame movement, color traces, and a flame flickering detection algorithm. For fire pixel detection, Celik et al. [110] used CIE L*a*b* color space. It does, however, have high false alarm proportions [111], which is only appropriate for short distances and major fires [112]. A basic flow process for fire pixel detection from the original image using RGB and YCbCr color models is given in Figure 8.

Only color information is not enough to obtain reliable results; the movement of fire is another element. Similar to other moving objects (such as walkers), their behavior is distinct. Different techniques are reported in the literature [113], such as the method of background subtraction, temporal differentiation, and optical flow analysis [114]. The motion features and YUV color model for fire detection were used by Marbach et al. [115]. Gunay et al. [116] used the Markov model to distinguish the flame motion from other objects with similar flame color, and the temporal wavelet analysis is used to detect the flame boundary. In addition, the authors have programmed an active learning function based on LMS. Toreyin et al. [117] used the temporal wavelet, spatial analysis, and heuristic thresholds for color and movement data for flame detection, which is impractical for fire hazards in real life. A system for fire-flame detection was proposed by Habiboglu et al. [118], where a mobile camera detects moving fire pixels without background removal. For each space-time block, they used time-based, color, and spatial information in feature vectors, but the system’s range of detection is very limited. Ko et al. [119] proposed flame detection using fuzzy finite automata, and this approach takes the irregularity of the flames into account. Wang et al. [120] used color and movement likelihood determination to form feature vectors for the detection of flame. The Wald–Wolfwitz algorithm is applied to feature vectors, and then convolution is used to improve the reliability. A stochastic method based on color and motion characteristics was proposed by Zhang et al. [121]. Foggia et al. [122] suggested a form of fire detection that uses a multi-expert system based on color, shape variation, and motion analysis. The relative change of the red, green, and blue pixels in the histogram-based strategy is another interesting warning. If the green pixel has a large standard deviation, it indicates that further validation is required [123]. 

Another function is the image frame’s dynamic texture analysis. This function can improve the precision of fire spotting, but the price of computing is higher, and a significant stage in video fire detection is edge detection. Dimitropoulos et al. [124] used dynamic texture analysis to detect flames in video. Qiu et al. [125] suggested a flame edge algorithm with steps, such as Sobel operator, gray level adjustment, smoothing, TH and TL adjustment, and PEI removal of irrelevant edges to obtain simple edges. A visualization technique for flame sensing through logistic feature regression was proposed by Kong et al. [111]. It has the advantages of being simple to construct and computationally light for real-time fire detection, and a slight loss of sensitivity is used to minimize false alarms by temporal smoothening. Chi et al. [126] used color, complex texture, and contour function for a wide range of almost 50 m of fire detection. Based on the fractal dimension, the fire contour is analyzed, and the gradient motion history picture is used to extract moving regions. Shen et al. [127] used YOLO’s flame detection model and compared it with methods of shallow learning. Moreover, it was proposed to incorporate other deep learning techniques, such as Boltzmann, auto-encoder and so on for potential improvements. 

### 3.4. Smoke Sensors

Smoke is emitted far earlier than other fire characteristics throughout the growth and development phases. In the initial phases of fire, quick smoke detection will increase the likelihood of effective fire suppression, successful firefighting, escape, and survival. Through making a light beam or electromagnetic radiation pass the interface of the particles, smoke can be detected. Smoke mass concentration, volume fraction, and size dissemination are known as primary smoke detection parameters. Smoke detectors must be able to respond to combustion and the smoke generated by flaming because there are significant differences in the structure and composition of the smoke generated by these fires [128]. The smoke produced by the burning flame is often larger than the combustion product particles. Fire creates smoke during combustion, which is a collection of solid particles, liquid particles, and gases in the air. It is created by material combustion and also reduces the air quality in the environment [129]. For smoke measurement, non-visual and visual techniques are used. They are summarized according to their characteristics as below. 

#### 3.4.1. Non-Visual Smoke Detection

Smoke measurement depends on the conditions of smoke combustion, for instance, pyrolysis, flaming, and smoldering. The method for detecting smoke is determined by the type of fire and its location. Smoke measurement technology based on photoelectric is specifically used for smoldering and can detect fire more quickly. The smoke is measured by the ionization level of the air by an ionization smoke sensor. The detection is done by creating a potential difference between the chambers and measuring the current that results. The ability of smoke to scatter light is determined by the amount of smoke in the air. By measuring the difference in light dispersion using an optical device, the photoelectric smoke sensor calculates the smoke level [11]. The ionization chamber smoke detector (ICSD) senses the fire generated when smoke particles reach the ionization chamber and alter the current by interfering with the ions’ flow. The movement of ions is influenced by temperature, pressure, gas composition, and moisture. One kind of ICSD with segregated ionization chambers has been designed to accomplish detection sensitivity; one chamber was completely sealed and not affected by the environment, while the other chamber was exposed to ambient air samples [130]. 

The multi-point induction detector, which combines smoke detection with other types of sensors, is seen to be a good option that can deliver a varied range of detection capabilities and minimize the irritant alarm without sacrificing the sensitivity of the smoke detector [131]. A multi-sensing detector was generated to detect both burning and smoldering flames by combining optical smoke detection with thermal sensing [132]. A substitute for ionization detectors is the integration of optical and thermal sensors with intellectual algorithms. Other multimodal sensing research includes detectors that combine photoelectric and gas sensing [133], ion and gas sensing [134], and photoelectric and ion with thermal sensing [135]. Gottuk et al. [136] presented multi-sensory smoke sensing methods, using various smoke measurement combinations, such as photoelectric-based gas sensing and ion-sensing gas sensing, to minimize false alarms. In an open door apartment, Jeong et al. [137] studied the smoke path and also built a fire field model to calculate the pace of fire spread. Liu et al. [138] suggested a smoke particle detector with an amorphous silicon film as the radiation source. A highly sensitive smoke detector was introduced by Bakhoum et al. [139] in which the alpha particles impact the MOSFET’s gate terminal and generate a positive charge. The surge in smoke particles in the region of the detector reduces the amount of alpha particles on the terminal of the gate, causing a reduction in the current that indicates a rise in the amount of smoke. Aspey et al. [140] devised a smoke optical detection device, composed of optical fiber, white polychromatic LED, Pyrex glass window and photodiode, to provide information on the burning materials. For wood smoke, the authors analyzed the transmission spectrum.

#### 3.4.2. Visual Smoke Detection

Smoke and fire both can be captured by cameras. The flow of smoke is quicker and presents usually at the beginning of a fire, but compared with flame detection, it is difficult to detect [141]. For flame and smoke detection, pixel rules for the values R, G, B, and models have been established based on different color spaces to show better results [142]. Gubbi et al. [143] detected smoke via the block approach by discrete cosine transforms and wavelets. In order to eliminate the false positives caused by the heuristic function, Ko et al. [144,145] suggested a luminance map and Bayesian network algorithm based fire sensing approach that supports the vector machines (SVM) algorithm. It needs a large number of frames, so the response time is long. In the video-based detection, Yuan et al. [146] used dynamic texture analysis to detect smoke. Qureshi et al. [147] developed a flame and smoke detection system based on color and motion cues. To enhance the system’s efficiency, they conducted morphological operations. Yuan et al. [148] merged Adaboost with a smoke detection staircase search technique and used dynamic smoke validity analysis to improve the effectiveness of smoke detection. This method for fire detection provides good performance if the trained dataset is quite big and algorithms are portable without sacrificing precision. 

**Figure 9:**
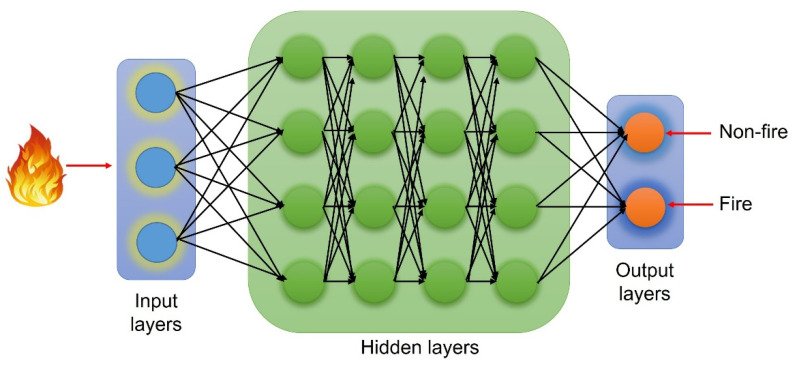
Schematic illustration of convolutional neural networks (CNNs) for fire detection.

Convolutional neural networks (CNNs) have enabled vision-based systems to detect fire and smoke during surveillance following recent developments in embedded processing [149,150]. It is one-of-a-kind in that it can efficiently recognize and understand patterns in images and feed-forward. Sergio et al. [151] proposed a real-time and embedded implementation of smoke detection technology that can be used with conventional surveillance video cameras using YOLOv2 CNN. Khan et al. [152] proposed an early fire detection framework for CCTV security cameras that use fine-tuned CNNs to detect smoke in a variety of interior and outdoor scenarios, and correspondingly proposed a model for hazy environments using semantic segmentation architecture [153]. Yakhyokhuja et al. [154] employed dilated convolutions to detect smoke, which was fully automated and the amount of false alarms was reduced due to generalization. Figure 9 shows a basic model of deep learning algorithm CNNs.

### 3.5. Multifarious Sensors

In the literature, additional fire detection technologies are mentioned. For fire and motion detection, Ruser et al. [155] presented an ultrasonic microwave multi-sensor fusion technique. The Doppler shift of an ultrasonic signal was used to assess the fluctuation in smoke density and heat. Schmitz et al. [156] proposed a sensor design based on the unique sensory process of fire measurement which is found in a specific type of insect. A sensor using a current loop circuit made up of numerous nickel wire segments was proposed by L’vov et al. [157]. Each section contains a voltage that can be evaluated using a microprocessor. Ishigaki et al. [158] suggested an approach to information fusion with only a single sensor. Each sensor is ideal for a specific variable to be sensed, nevertheless, it is also vulnerable to a slew of other factors that can act as disturbance or noise signals. In order to achieve more precise fire detection, a novel way of detecting fire was tested using microholography by Hai et al. [159]. The microscopic holographic technology is capable of detecting three-dimensional models of fire smoke particles. Recently, Zhang et al. [160] have presented a dynamic model for fire detection based on triboelectric nanogenerators and fluid-dynamic modeling that can capture multi-directional breeze energy and be self-powered. The following are the various types of miscellaneous approaches that have been reported for fire detection. 

#### 3.5.1. Microwave Radiometers for Fire Detection

Under severe conditions, microwave radiometers can also be used for fire detection. Sensors based on these technologies, in the presence of smoke, vapors, and dust work suitably. These strategies are appropriate for fire sensing in open regions, such as forests. A Ku band radiometer prototype for forest fire findings was suggested by Bianchi et al. [161]. The current studies in this field were further addressed by Dvorak et al. [162]. The significance of the parameters of fire spot emissivity, which was not found in previous studies, was discussed, and measurements were made for various fire types.

#### 3.5.2. Acoustic Wave Fire Detection

Surface acoustic wave sensors can also be used in harsh environments for fire detection. They are small in size and powerful and can work under conditions of variable frequency and high bandwidth. These sensors are wireless, passive, and less radiation-affected. Therefore, they are useful for other fields, such as industry and aerospace. However, during resonant frequency measurement, these sensors have a number of disadvantages. An algorithm to correct these errors was proposed by Liu et al. [163]. Beisner et al. [164] suggested sound wave-based fire elimination research and found that the sound waves at 30.6 Hz are ideal frequency waves for rapidly stopping the flames. Salauddin et al. [165] developed a space station fire extinguishing system based on sound waves.

#### 3.5.3. Deep Learning for Fire Detection

Deep learning is used to handle challenging problems in the realm of digital image processing, such as picture colorization, classification, segmentation, and detection, which are the core aspects of fire and smoke detection. Deep learning technology incorporates a nonlinear and complex model abstraction transformation into a vast database. Hong et al. [166] presented a combination of machine learning, as well as an adaptive fuzzy algorithm, with a fire detection accuracy of above 95%. The model contains five convolution layers and one fully-connected layer that learns two classes of fire situations and warning situations. The Qin et al. [167] model classified fire images using depthwise separable convolution and YOLOv3 for target classification and position regression; detection accuracy was 98% with a detection rate of 38 frames per second. Using large fire dataset imagery to generate accurate predictions, Avazov et al. [168] developed an enhanced YOLOv4 algorithm that accurately detects even little sparks in varied weather conditions and sounds an alarm within 8 s of a fire outbreak. The multimodality fusion approach suggested by Ren et al. [169] was efficient in identifying electric fires by locating the arc fault that causes electrical fires in the buildings’ low-voltage distribution system. In comparison to earlier studies, Park et al. [170] proposed an algorithm utilizing ELASTIC-YOLOv3, temporal fire-tube, and histograms for real-time fire detection at night in urban areas, which demonstrated good results at night. Deep learning has pushed the boundaries of what was previously thought to be achievable in the field of digital image processing. 

#### 3.5.4. Fire Fighting Robot System

The fire detecting system based on immovable sensors has limitations. Under harsh conditions, the mobility rendered to the fire sensors makes them comparatively secure. These limitations can be overcome by deploying fire sensors and extinguishers deployed robots. In order to detect and fight fire, robots can travel on the ground or fly. Causalities and dangers of firefighters may be reduced in this way. In order to make lightweight robots, research is being carried out so that it is easier for them to fly. Liu et al. [171] addressed the ongoing research in this field and identified the various characteristics of current firefighting robots as well as the sensors they employ. Ando et al. [172] suggested flying a lightweight robot-style hose propelled by a water jet near the fire source. To control the trajectory of emissions, it contains a nozzle module and motors on the top. A hydraulic based firefighting snake robot was built by Liljeback et al. [173], and its applications and design challenges were addressed. Researchers at the Italian Institute of Technology have created a walk-man firefighting robot, which has a human form and is capable of carrying up to 10 kg of weight and lifting a variety of goods [174]. LUF technology devised a ground vehicle robot (LUF 60) for outdoor firefighting. Smoke reduction, stair climbing and fire extinguishing are the characteristics defined for this robot, and they stated the capacity to throw water up to 80 m in height at a rate of 800 GPM [175]. Parosha group designed another firefighting robot vehicle, that has feathers of laser range finders, thermal imagers, and an acoustic detection system [176]. The Control Farayand Pasargad (CFP) group manufactured a turbine aided firefighting robot that has feathers of smoke reduction, obstacle clearance, remote activity, and fire extinguishing [177]. Howe and Howe technologies created the Thermite RS1-T3 robot [178]. High-definition analog camera and infrared FLIR were used by the robot to acquire fire data, and it has a huge water distribution capability of 1250 GPM. Firemote is a UGV fire robot made by the Ryland research team [178]. The function is that it operates and distributes water and foam to extinguish a fire in hazardous areas. Archibot-m is a water-resistant firefighting robot produced by DRB fate Ltd., that detects fire using a visual camera. The most remarkable feature of this robot is that it is suitable for indoor firefighting operations and effective stair climbing [179]. The demolition process is also required in the fire threat region to clear the escape path. Brock is a robot, specially designed for narrow working areas (such as climbing stairs) designed by the Brokk company [180]. Fire extinguishment is the foremost objective of these firefighting robots. Water, chemicals, foam, and CO_2_ are used by traditional fire extinction techniques, however, in dangerous scenarios, their use will cause issues.

## 4. Conclusions and Perspective

Several innovative fire sensing systems developed in the past decade possess the exceptional potential to decrease false alarms, improve fire sensitivity and quick response, and increase fire safety. This section provides an overview of the research that has been performed in the field of fire detecting technology. It focuses on sensor actuator-based fire detection systems for buildings. It outlines the shortcomings and limitations of existing fire sensing systems and provides suggestions for essential changes. The basic goal of a fire detection system is to identify fire early, with as few false alarms as possible. A quick fire detection system necessitates a sensor with a quick response time that can sense fire threats in their early stages.

Pre-flashover and post-flashover periods have often been used to split the course of a compartment fire. It is stated that understanding the course of a fire before it flashes over is critical for saving lives, and understanding its characteristics after it flashes over gives a foundation for creating property-saving measures. During a rescue, PEFs evacuation conditions put both firefighters and trapped persons in grave danger, which may be successfully dealt with using BIM.

Heat sensing systems are more reliable and provide fewer false alarms, but they are sluggish to react. By mobilizing these heat sensors, their response time can be enhanced. Heat sensors are usually mounted on the walls or ceilings, and fire spreads mostly through the flooring. Optical heat detectors that use a change in refractive index are highly sensitive to even modest temperature changes. It is important to conduct research into their application in fire detection systems, and their light weight and low-power units must be able to meet the latest wireless sensor network requirements. Optical heat sensors are widely used in mines and tunnels, according to the majority of the literature. However, they are ideal for use in environments with galleries, big halls, and complex spaces. It can replace and minimize the budget of using a number of point heat sensors in a building. Direct automated actuation signal for valves utilized in fire extinguishing systems is ideal for bi-metal style heat sensors. Thermal sensors based on the rate of heat change are ideal for fire detecting applications since an absolute temperature value is inadequate to sense a fire danger. However, change rate data can be obtained from any non-rate type of thermal sensor by altering the algorithm employed on the output signal of the thermal sensor. It is important because non-rate heat sensors are quicker to respond than rate style heat sensors. The thermistor is a small, light, and responsive temperature sensor. In the literature, better linearization algorithms are provided that can be put in sensor arrays and are appropriate for fire localization. However, further research is needed to evaluate their feedback for various shape arrays. These sensors are preferred over conventional thermal sensors with a bulkier mechanical structure.

A smoke sensor has a high false alarm, but the combination of visual sensing systems can increase its performance. Different sensors produce different signal formats, and effective signal conditioning is required for their interaction with other sections of the fire detection system. The existing signal conditioning hardware and algorithms are inadequate and there is a lot of room for improvement.

The main issues with most gas sensors are irreversibility, volatility, and low selectivity, and as a result, their usage in buildings is restricted. Metal oxide semiconductor gas sensors have high sensitivity and are inexpensive. However, their inability to maintain equilibrium necessitates temperature compensation. Since temperature usually varies during a fire hazard, this aspect becomes even more significant, because it has the potential to reduce the productivity of gas sensors. Hence, it is necessary to carry out experimental research on the temperature recompense of CO, CO_2_, HCN, and other fire dangerous gases produced during fire. Nonlinearity is another issue with metal oxide semiconductor gas sensors. As a result, compensation in the form of hardware or software is also required to account for this aspect. The gas sensor based on carbon nanotubes has a high sensitivity, is less corrosive, and has a low cost, short response time, strong adsorptive capability, and a wide bandwidth. Optical gas sensors are highly sensitive, selective, and reliable but they have heavy weight and are more expensive. Further study is needed to minimize their price and weight for application in smart constructions.

Non-visual fire sensing methods are supplemented by vision-based fire, flame and smoke detecting approaches. Both visible and infrared camera detecting approaches have benefits and limitations. Near-infrared cameras are less expensive, but they have a limited range. Thermal distance issues, IR blocking, and thermal reflection are all disadvantages of IR techniques.

Deep learning allows for better accuracy in fire detection, including picture classification, semantic segmentation, object detection, and simultaneous location when compared to traditional computer vision techniques. Deep learning applications require less expert analysis and fine-tuning since neural networks are trained rather than programmed, allowing them to take advantage of the massive amounts of video and image data available in today’s systems.

The use of robots in the detection of fires has opened up a new research field. The firefighting robots will supplement the work of human firefighters, while they cannot take their place since human intellect surpasses that of robots. However, if there is a major fire hazard, human life cannot be put in jeopardy, and using robots is a safer choice. They could either be self-contained or governed by humans on the outside. The importance of balancing when running cannot be overstated. In this regard, robots are currently not up to par with humans. The fire extinguishing and sensor systems mounted on them make them heavier, which makes balancing and reaching high speeds difficult. This is a significant issue for indoor fire detection systems. At present, the emphasis is on using robots to extinguish fires in emergency situations, and, in the vast majority of situations, this procedure is carried out from outside the burning building.

In the fire sensing system based on a wireless sensor network (WSN), detectors are placed as detector nodes with built-in communication hardware. The key problem is to make them low-power, stable, and error tolerant. The flame sensor has a high false alarm due to infrared, visible, and ultraviolet radiation. These interferences are caused by non-fire sources and need to be compensated. EMI/RFI noise will also affect the performance of the sensor, so it needs to be studied.

Fire detection and control is a complicated operation. Because of its various phases, diverse appearance, colors, emission spectra, combustion fuel, and position, complexities arise. In these circumstances, using fuzzy logic and deep learning-based algorithms to improve the current fire detection system’s performance could be beneficial. Optimization techniques need to be improved to minimize false alarms. After simultaneous interpreting, all data from various sensors must be processed and analyzed according to sensor fusion technology.

## Figures and Tables

**Table 1:** Recent developments and comparison of different heat sensors and their characteristics.

Sensor	Detection Element	Construction and Working Principle	Response Time	Detection Area	Features and Advantages	Ref.
Distributed Optical Fiber Heat Detectors	Two parallel optical fibers	By measuring the temperature of hot air flows	40 s	Wide ranges	Simple and efficient	[56]
	Graphene-coated optical fiber	Fiber Bragg grating	18-fold faster than conventional fiber heat detectors	1 km	Long-distance and fast optical transmission	[57]
	Multi-core fiber	Raman scattering	Real-time	10 km	Self-calibration	[58]
Thermal Resistance Sensors	Ammonium polyphosphate and GO	Freeze-drying	~2.6 s	Small	Compressible	[59]
	FGO/CNTs	Layer-by-layer	5 s	Small	Twisted and bended	[60]
	AgNW/FPVB and GO/FC	Spray coating	0.83 s	Large (>30 cm)	Hydrophobic and self-cleaning	[47]
	MPMS and LLA	EISA	∼1 s	Small	Twisted, folded and Structure stability	[61]
	RGOP-NaCl	Evaporation-induced self-assembly	5.3 s	Small	Twisted, can fuse function and can cut off in fire	[62]
	GO-BA	Evaporation-induced self-assembly	∼0.8 s	Small	Twisted and bended	[63]
	APP/GO/TFTS	Water-based coating	2 s	Small	Flexible and Super-hydrophobic	[64]
	MPTS-GO	TEISA	1 s	Small	Twisted and bended	[65]
	CCS/MMT/A-CNT	Freeze-drying	~0.25 s	Small	Light weight and Compressible	[66]
Miscellaneous Heat Detectors	Thermistor	Steinhart-Hart equation	260 s	Small	Suitable for sprinklers	[67]
	Bi-spectrum camera	YOLOv3 and TNNI	0.6 s	Limited to camera vision	Low cost, and automatic disposal of devices	[68]
	Thermocouple anddigital multimeter	Operational algorithm	2.3 times faster	Small	Useful where temperaturevaries	[69]
	Artificial intelligence	LSTM and TCNN	1 s	5 m	Predict fire danger before 60 s	[70]
	Rate of temperature rise	Operational algorithm	120–180 s	Small	Useful where temperature varies	[71]

Note: FGO: functionalized graphene oxide; AgNW: silver nanowire; FPVB: fluoride polyvinyl butyral; FC: functional cellulose; MPMS: 3-methacryloxypropyltrimethoxysilane; LAA: L-ascorbic acid; EISA: evaporation-induced self-assembly process; CCS: carboxymethyl chitosan; MMT: montmorillonite; A-CNT: amino-functionalized carbon nanotube; RGOP: reduced graphene oxide paper; TFTS: tetra hydroperfluorodecyltrimethoxy silane; TEISA: low-temperature evaporation-induced self-assembling; LSTM: long short-term memory; TCNN: transpose convolution neural network; TNNI: turing neural network inference.

## References

[1] Brushlinsky P.W.N., Ahrens M., Sokolov S. (2019). World Fire Statistics. https://www.ctif.org/.

[2] Joo J.-Y., Ilić M.D. (2016). An information exchange framework utilizing smart buildings for efficient microgrid operation. Proc. IEEE.

[3] Morgan A. (2000). New Fire Detection Concepts. Fire Saf. Eng..

[4] Liu Z., Makar J., Kim A.K. (2001). Development of fire detection systems in the intelligent building. NIST Spec. Publ. SP.

[5] Crapo W.R. (2002). Smoke Detectors and Life Safety. Fire Eng..

[6] Purser D.A. (2016). Toxicity Assessment of Combustion Products. The SFPE Handbook of Fire Protection Engineering.

[7] Fonollosa J., Solórzano A., Marco S. (2018). Chemical sensor systems and associated algorithms for fire detection: A review. Sensors.

[8] Gaur A., Singh A., Kumar A., Kumar A., Kapoor K. (2020). Video flame and smoke based fire detection algorithms: A literature review. Fire Technol..

[9] Çetin A.E., Dimitropoulos K., Gouverneur B., Grammalidis N., Günay O., Habiboǧlu Y.H., Töreyin B.U., Verstockt S. (2013). Video fire detection–Review. Digit. Signal Process..

[10] Kaabi R., Frizzi S., Bouchouicha M., Fnaiech F., Moreau E., R Kaabi S.F., Moreau E. Video Smoke Detection Review: State of the Art of Smoke Detection in Visible and IR Range. Proceedings of the 2017 International Conference on Smart, Monitored and Controlled Cities (SM2C).

[11] Bogue R. (2013). Sensors for fire detection. Sens. Rev..

[12] Ghali R., Jmal M., Mseddi W.S., Attia R. Recent Advances in Fire Detection and Monitoring Systems: A Review. Proceedings of the International conference on the Sciences of Electronics, Technologies of Information and Telecommunications.

[13] Barmpoutis P., Papaioannou P., Dimitropoulos K., Grammalidis N. (2020). A review on early forest fire detection systems using optical remote sensing. Sensors.

[14] Ukil A., Braendle H., Krippner P. (2011). Distributed temperature sensing: Review of technology and applications. IEEE Sens. J..

[15] Garlock M., Paya-Zaforteza I., Kodur V., Gu L. (2012). Fire hazard in bridges: Review, assessment and repair strategies. Eng. Struct..

[16] Debliquy M., Lahem D., Bueno-Martinez A., Ravet G., Renoirt J.-M., Caucheteur C. (2015). Review of the Use of the Optical Fibers for Safety Applications in Tunnels and Car Parks: Pollution Monitoring, Fire and Explosive Gas Detection. Sensing Technology: Current Status and Future Trends III.

[17] Kennedy P.M., Kennedy K.C., Kennedy J.A. (2003). Flashover and Fire Analysis—A Discussion of the Practical Use of Flashover Analysis in Fire Investiga-tions.

[18] Harmathy T.Z., Mehaffey J.R. (1983). Post-Flashover compartment fires. Fire Mater..

[19] Wickström U. (2016). Temperature Calculation in Fire Safety Engineering.

[20] Notarianni K.A., Cyganski D., Duckworth R.J. Development of a Portable Flashover Predictor (Fire-Ground Environment Sensor System). Proceedings of the International Conference on Safety (ICS2012).

[21] Fang H., Lo S.M., Zhang Y., Shen Y. (2021). Development of a machine-learning approach for identifying the stages of fire development in residential room fires. Fire Saf. J..

[22] He Y., Wang J., Wu Z., Hu L., Xiong Y., Fan W. (2002). Smoke venting and fire safety in an industrial warehouse. Fire Saf. J..

[23] Feasey R., Buchanan A. (2002). Post-flashover fires for structural design. Fire Saf. J..

[24] Behnam B. (2017). Post-Earthquake Fire Analysis in Urban Structures: Risk Management Strategies.

[25] Khorasani N.E., Garlock M.E.M. (2017). Overview of fire following earthquake: Historical events and community responses. Int. J. Disaster Resil. Built Environ..

[26] Wang S.-H., Wang W.-C., Wang K.-C., Shih S.-Y. (2015). Applying building information modeling to support fire safety management. Autom. Constr..

[27] Lu X., Yang Z., Xu Z., Xiong C. (2020). Scenario simulation of indoor post-earthquake fire rescue based on building information model and virtual reality. Adv. Eng. Softw..

[28] Lotfi N., Behnam B., Peyman F. (2021). A BIM-based framework for evacuation assessment of high-rise buildings under post-earthquake fires. J. Build. Eng..

[29] Luan N., Ding C., Yao J. (2016). A refractive index and temperature sensor based on surface plasmon resonance in an exposed-core microstructured optical fiber. IEEE Photonics J..

[30] Jevtić R.B., Blagojević M.Đ. (2014). On a linear fire detection using coaxial cables. Therm. Sci..

[31] Wu J., Wu Z., Ding H., Wei Y., Yang X., Li Z., Yang B.-R., Liu C., Qiu L., Wang X. (2019). Multifunctional and high-sensitive sensor capable of detecting humidity, temperature, and flow stimuli using an integrated microheater. ACS Appl. Mater. Interfaces.

[32] Hoff H. Using Distributed Fibre Optic Sensors for Detecting Fires and Hot Rollers on Conveyor Belts. Proceedings of the 2017 2nd International Conference for Fibre-Optic and Photonic Sensors for Industrial and Safety Applications (OFSIS).

[33] Barrias A., Casas J.R., Villalba S. (2016). A review of distributed optical fiber sensors for civil engineering applications. Sensors.

[34] Wang A., Liu W., Li X., Yue C., Wang Y., Wang Q., Cai X. Distributed optical fiber temperature detecting and alarm system. Proceedings of the 12th International Conference on Automatic Fire Detection.

[35] Yilmaz G., Karlik S.E. (2006). A distributed optical fiber sensor for temperature detection in power cables. Sens. Actuators A Phys..

[36] Laarossi I., Quintela-Incera M.Á., López-Higuera J.M. (2019). Comparative experimental study of a high-temperature raman-based distributed optical fiber sensor with different special fibers. Sensors.

[37] Meacham B.J. (1994). International developments in fire sensor technology. J. Fire Prot. Eng..

[38] Bao X., Brown A., DeMerchant M., Smith J. (1999). Characterization of the Brillouin-loss spectrum of single-mode fibers by use of very short (<10-ns) pulses. Opt. Lett..

[39] Liu Z., Ferrier G., Bao X., Zeng X., Yu Q., Kim A.K. (2003). Brillouin scattering based distributed fiber optic temperature sensing for fire detection. Fire Saf. Sci..

[40] Junwei D., Weiping L., Cui T., Xida Y. (2020). Fire detector based on serial FBG temperature sensors optical cabling. J. Phys. Conf. Ser..

[41] Wang J., Li Z., Fu X., Wang H., Jiang D. (2021). Distributed temperature sensing system based on a densely spaced FBG array for small fire recognition. Measurement.

[42] Zhu Y., Klegseth M., Bao Y., Hoehler M.S., Choe L., Chen G. (2021). Distributed fiber optic measurements of strain and temperature in long-span composite floor beams with simple shear connections subject to compartment fires. Fire Saf. J..

[43] Ju H.-M., Huh S.H., Choi S.-H., Lee H.-L. (2010). Structures of thermally and chemically reduced graphene. Mater. Lett..

[44] Khan F., Wang S., Ma Z., Ahmed A., Song P., Xu Z., Liu R., Chi H., Gu J., Tang L.C. (2021). A Durable, Flexible, Large-Area, Flame-Retardant, Early Fire Warning Sensor with Built-In Patterned Electrodes. Small Methods.

[45] Wu Q., Gong L.-X., Li Y., Cao C.-F., Tang L.-C., Wu L., Zhao L., Zhang G.-D., Li S.-N., Gao J. (2017). Efficient flame detection and early warning sensors on combustible materials using hierarchical graphene oxide/silicone coatings. ACS Nano.

[46] Xie H., Lai X., Li H., Gao J., Zeng X., Huang X., Lin X. (2019). A highly efficient flame retardant nacre-inspired nanocoating with ultrasensitive fire-warning and self-healing capabilities. Chem. Eng. J..

[47] Xie H., Lai X., Li H., Gao J., Zeng X., Huang X., Zhang S. (2019). A sandwich-like flame retardant nanocoating for supersensitive fire-warning. Chem. Eng. J..

[48] Xu H., Li Y., Huang N.-J., Yu Z.-R., Wang P.-H., Zhang Z.-H., Xia Q.-Q., Gong L.-X., Li S.-N., Zhao L. (2019). Temperature-triggered sensitive resistance transition of graphene oxide wide-ribbons wrapped sponge for fire ultrafast detecting and early warning. J. Hazard. Mater..

[49] Huang N.-J., Cao C.-F., Li Y., Zhao L., Zhang G.-D., Gao J.-F., Guan L.-Z., Jiang J.-X., Tang L.-C. (2019). Silane grafted graphene oxide papers for improved flame resistance and fast fire alarm response. Compos. Part B Eng..

[50] Qu Z., Xu C.A., Li X., Wu Y., Wang K., Zheng X., Cui X., Wu X., Shi J., Wu K. (2021). Facile Preparation of BP-MoS2/GO Composite Films with Excellent Flame Retardancy and Ultrasensitive Response for Smart Fire Alarm. Chem. Eng. J..

[51] Qu Z., Wu K., Xu C.-A., Li Y., Jiao E., Chen B., Meng H., Cui X., Wang K., Shi J. (2021). Facile Construction of a Flexible Film with Ultrahigh Thermal Conductivity and Excellent Flame Retardancy for a Smart Fire Alarm. Chem. Mater..

[52] Chiang C.-T., Chang F.-W. (2015). Design of a calibrated temperature difference sensor transducer for monitoring environmental temperature difference applications. IEEE Sens. J..

[53] Jevtić R.B., Blagojević M.Đ. Linear fire detection with distance determination using coaxial cables. Proceedings of the 2011 19th Telecommunications Forum (TELFOR) Proceedings of Papers.

[54] Wang S., Berentsen M., Kaiser T. (2005). Signal processing algorithms for fire localization using temperature sensor arrays. Fire Saf. J..

[55] Bosch I., Gomez S., Molina R., Miralles R. Object discrimination by infrared image processing. Proceedings of the International Work-Conference on the Interplay between Natural and Artificial Computation.

[56] Sun M., Tang Y., Yang S., Li J., Sigrist M.W., Dong F. (2016). Fire source localization based on distributed temperature sensing by a dual-line optical fiber system. Sensors.

[57] Guo Y., Han B., Du J., Cao S., Gao H., An N., Li Y., An S., Ran Z., Lin Y. (2021). Kilometers Long Graphene-Coated Optical Fibers for Fast Thermal Sensing. Research.

[58] Du H., Wu H., Zhang Z., Zhao C., Zhao Z., Tang M. (2021). Single-ended self-calibration high-accuracy Raman distributed temperature sensing based on multi-core fiber. Opt. Express.

[59] Cao C., Yuan B. (2021). Thermally induced fire early warning aerogel with efficient thermal isolation and flame-retardant properties. Polym. Adv. Technol..

[60] Chen W., Liu P., Liu Y., Wang Q., Duan W. (2018). A temperature-induced conductive coating via layer-by-layer assembly of functionalized graphene oxide and carbon nanotubes for a flexible, adjustable response time flame sensor. Chem. Eng. J..

[61] Zhang Z.-H., Zhang J.-W., Cao C.-F., Guo K.-Y., Zhao L., Zhang G.-D., Gao J.-F., Tang L.-C. (2020). Temperature-responsive resistance sensitivity controlled by L-ascorbic acid and silane co-functionalization in flame-retardant GO network for efficient fire early-warning response. Chem. Eng. J..

[62] Chen G., Yuan B., Zhan Y., Dai H., He S., Chen X. (2020). Functionalized graphene paper with the function of fuse and its flame-triggered self-cutting performance for fire-alarm sensor application. Mater. Chem. Phys..

[63] Yuan B., Wang Y., Chen G., Yang F., Zhang H., Cao C., Zuo B. (2021). Nacre-like graphene oxide paper bonded with boric acid for fire early-warning sensor. J. Hazard. Mater..

[64] Guo K.-Y., Wu Q., Maoa M., Chenb H., Zhanga G.D., Zhaoa L., Gaoc J.F., Songd P., Tang L.-C. (2020). Water-based hybrid coatings toward mechanically flexible, super-hydrophobic and flame-retardant polyurethane foam nanocomposites with high-efficiency and reliable fire alarm response. Compos. Part B Eng..

[65] Huang N.-J., Xia Q.-Q., Zhang Z.-H., Zhao L., Zhang G.-D., Gao J.-F., Tang L.-C. (2020). Simultaneous improvements in fire resistance and alarm response of GO paper via one-step 3-mercaptopropyltrimethoxysilane functionalization for efficient fire safety and prevention. Compos. Part A Appl. Sci. Manuf..

[66] Chen J., Xie H., Lai X., Li H., Gao J., Zeng X. (2020). An ultrasensitive fire-warning chitosan/montmorillonite/carbon nanotube composite aerogel with high fire-resistance. Chem. Eng. J..

[67] Qualey J.R., Desmarais L., Pratt J. (2001). Response-time comparisons of ionization and photoelectric/heat detectors. NIST Spec. Publ. SP.

[68] Ma Y., Feng X., Jiao J., Peng Z., Qian S., Xue H., Li H. (2020). Smart Fire Alarm System with Person Detection and Thermal Camera. International Conference on Computational Science.

[69] Kushnir A., Kopchak B., Gavryliuk A. (2021). Operational Algorithm for a Heat Detector Used in Motor Vehicles. East.-Eur. J. Enterp. Technol..

[70] Wu X., Zhang X., Huang X., Xiao F., Usmani A. (2021). A Real-Time Forecast of Tunnel Fire Based on Numerical Database and Artificial Intelligence. Build. Simul..

[71] Schmoetzer K. (2001). Aircraft Fire Detection: Requirements, Qualification, and Certification Aspects. 12th International Conference on Automatic Fire Detection.

[72] Jackson M.A., Robins I. (1994). Gas sensing for fire detection: Measurements of CO, CO_2_, H_2_, O_2_, and smoke density in European standard fire tests. Fire Saf. J..

[73] Barrett R. (2000). CO Fire Detection: A Useful Technique? Report on a Test Programme and Discusses the Issues Involved in the Use of CO Fire Detectors. Fire Saf. Eng..

[74] Liu X., Cheng S., Liu H., Hu S., Zhang D., Ning H. (2012). A survey on gas sensing technology. Sensors.

[75] Wiedinmyer C., Neff J.C. (2007). Estimates of CO2 from fires in the United States: Implications for carbon management. Carbon Balance Manag..

[76] Stec A.A. (2017). Fire toxicity–The elephant in the room?. Fire Saf. J..

[77] Reddy A.P.K., Reddy E.S., Bhaskar T., Yadav B.P., Singh A.K. (2020). Design of Fire and Gas Detection System for a Process Plant: A Review. Advances in Industrial Safety.

[78] Jabłoński K., Grychowski T. The Method for Easy Identifying Zero Temperature Drift of Catalytic Bead Sensor. Proceedings of the 2018 XV International Scientific Conference on Optoelectronic and Electronic Sensors (COE).

[79] Mann D.P., Pratt K.F.E., Paraskeva T., Parkin I.P., Williams D.E. (2007). Transition metal exchanged zeolite layers for selectivity enhancement of metal-oxide semiconductor gas sensors. IEEE Sens. J..

[80] Naik A., Parkin I., Binions R. (2016). Gas sensing studies of an NN hetero-junction array based on SnO_2_ and ZnO composites. Chemosensors.

[81] Prajapati C.S., Soman R., Rudraswamy S.B., Nayak M., Bhat N. (2017). Single chip gas sensor array for air quality monitoring. J. Microelectromech. Syst..

[82] Zhang L., Rahimabady M., Tan S.Y., Tan C.Y., Chen S., Chen Y.F., Yao K., Humbert A., Soccol D., Zang K. (2017). P (VDF-HFP) polymer as sensing material for capacitive carbon dioxide sensors. IEEE Sens. J..

[83] Riches J., Chapman A., Beardon J. The Detection of Fire Precursors Using Chemical Sensors. Proceedings of the 8th International Fire Science and Engineering Conference.

[84] Mandayo G.G., Castano E., Gracia F.J. (2002). Carbon monoxide detector fabricated on the basis of a tin oxide novel doping method. IEEE Sens. J..

[85] Juang F.-R., Fang Y.-K., Chiang Y.-T., Chou T.-H., Lin C.-I., Lin C.-W., Liou Y.-W. (2010). Comparative study of carbon monoxide gas sensing mechanism for the LTPS MOS Schottky diodes with various metal oxides. IEEE Sens. J..

[86] Adib M., Eckstein R., Hernandez-Sosa G., Sommer M., Lemmer U. (2017). SnO_2_ nanowire-based aerosol jet printed electronic nose as fire detector. IEEE Sens. J..

[87] Mirzaei A., Lee J.-H., Majhi S.M., Weber M., Bechelany M., Kim H.W., Kim S.S. (2019). Resistive gas sensors based on metal-oxide nanowires. J. Appl. Phys..

[88] Ebnali-Heidari M., Koohi-Kamali F., Ebnali-Heidari A., Moravvej-Farshi M.K., Kuhlmey B.T. (2014). Designing tunable microstructure spectroscopic gas sensor using optofluidic hollow-core photonic crystal fiber. IEEE J. Quantum Electron..

[89] Dankner Y., Jacobson E., Goldenberg E., Pashin S. (1995). Optical-Based UV-IR Gas Detector for Environmental Monitoring of Flammable Hydrocarbons and Toxic Gases. Environ. Monit. Hazard. Waste Site Remediat..

[90] Leis J., Buttsworth D. (2017). A robust method for tuning photoacoustic gas detectors. IEEE Trans. Ind. Electron..

[91] Zhang C., Yang Y., Tan Y., Ho H.L., Jin W. (2018). All-optical fiber photoacoustic gas sensor with double resonant enhancement. IEEE Photonics Technol. Lett..

[92] Gatsa O., Combette P., Rosenkrantz E., Fourmentel D., Destouches C., Ferrandis J.-Y. (2018). High-temperature ultrasonic sensor for fission gas characterization in MTR harsh environment. IEEE Trans. Nucl. Sci..

[93] Shi M., Bermak A., Chandrasekaran S., Amira A., Brahim-Belhouari S. (2008). A committee machine gas identification system based on dynamically reconfigurable FPGA. IEEE Sens. J..

[94] Kumar A., Hancke G.P. (2014). An energy-efficient smart comfort sensing system based on the IEEE 1451 standard for green buildings. IEEE Sens. J..

[95] Serio M.A., Bonanno A.S., Newman J.S. FT-IR Based System for Fire Detection. Proceedings of the NIST Annual Conference on Fire Research.

[96] Serio M.A., Bonamno A.S., Knight K.S., Newman J.S. Fourier Transform Infrared Diagnostics for Improved Fire Detection Systems. Proceedings of the NIST Annual Conference on Fire Research.

[97] Cleary T., Ono T. (2001). Enhanced Residential Fire Detection by Combining Smoke and CO Sensors (SP 965), Special Publication (NIST SP).

[98] Qiu X., Wei Y., Li N., Guo A., Zhang E., Li C., Peng Y., Wei J., Zalng Z. (2019). Development of an early warning fire detection system based on a laser spectroscopic carbon monoxide sensor using a 32-bit system-on-chip. Infrared Phys. Technol..

[99] Parent G., Acem Z., Lechêne S., Boulet P. (2010). Measurement of infrared radiation emitted by the flame of a vegetation fire. Int. J. Therm. Sci..

[100] Sidey J., Mastorakos E., Gordon R.L. (2014). Simulations of autoignition and laminar premixed flames in methane/air mixtures diluted with hot products. Combust. Sci. Technol..

[101] Xu L., Yan Y. (2006). A new flame monitor with triple photovoltaic cells. IEEE Trans. Instrum. Meas..

[102] Pauchard A.R., Manic D., Flanagan A., Besse P.A., Popovic R.S. (2000). A method for spark rejection in ultraviolet flame detectors. IEEE Trans. Ind. Electron..

[103] de Iacovo A., Venettacci C., Colace L., Scopa L., Foglia S. (2017). PbS colloidal quantum dot visible-blind photodetector for early indoor fire detection. IEEE Sens. J..

[104] Prema C.E., Vinsley S.S., Suresh S. (2018). Efficient flame detection based on static and dynamic texture analysis in forest fire detection. Fire Technol..

[105] Töreyin B.U., Cinbis R.G., Dedeoglu Y., Cetin A.E. (2007). Fire detection in infrared video using wavelet analysis. Opt. Eng..

[106] Celik T., Demirel H. (2009). Fire detection in video sequences using a generic color model. Fire Saf. J..

[107] Kozeki D. Smoldering Fire Detection by Image-Processing. Proceedings of the 12th International Conference on Automatic Detection.

[108] Khalil A., Rahman S.U., Alam F., Ahmad I., Khalil I. (2021). Fire Detection Using Multi Color Space and Background Modeling. Fire Technol..

[109] Chen J., He Y., Wang J. (2010). Multi-feature fusion based fast video flame detection. Build. Environ..

[110] Celik T. (2010). Fast and efficient method for fire detection using image processing. ETRI J..

[111] Kong S.G., Jin D., Li S., Kim H. (2016). Fast fire flame detection in surveillance video using logistic regression and temporal smoothing. Fire Saf. J..

[112] Muhammad K., Ahmad J., Mehmood I., Rho S., Baik S.W. (2018). Convolutional neural networks based fire detection in surveillance videos. IEEE Access.

[113] Torabian M., Pourghassem H., Mahdavi-Nasab H. (2021). Fire Detection Based on Fractal Analysis and Spatio-Temporal Features. Fire Technol..

[114] Mueller M., Karasev P., Kolesov I., Tannenbaum A. (2013). Optical flow estimation for flame detection in videos. IEEE Trans. Image Processing.

[115] Marbach G., Loepfe M., Brupbacher T. (2006). An image processing technique for fire detection in video images. Fire Saf. J..

[116] Günay O., Taşdemir K., Töreyin B.U., Çetin A.E. (2010). Fire detection in video using LMS based active learning. Fire Technol..

[117] Töreyin B.U., Dedeoğlu Y., Güdükbay U., Cetin A.E. (2006). Computer vision based method for real-time fire and flame detection. Pattern Recognit. Lett..

[118] Habiboğlu Y.H., Günay O., Çetin A.E. (2012). Covariance matrix-based fire and flame detection method in video. Mach. Vis. Appl..

[119] Ko B.C., Ham S.J., Nam J.Y. (2011). Modeling and formalization of fuzzy finite automata for detection of irregular fire flames. IEEE Trans. Circuits Syst. Video Technol..

[120] Wang D., Cui X., Park E., Jin C., Kim H. (2013). Adaptive flame detection using randomness testing and robust features. Fire Saf. J..

[121] Zhang Z., Shen T., Zou J. (2014). An improved probabilistic approach for fire detection in videos. Fire Technol..

[122] Foggia P., Saggese A., Vento M. (2015). Real-time fire detection for video-surveillance applications using a combination of experts based on color, shape, and motion. IEEE Trans. Circuits Syst. Video Technol..

[123] Borges P.V.K., Izquierdo E. (2010). A probabilistic approach for vision-based fire detection in videos. IEEE Trans. Circuits Syst. Video Technol..

[124] Dimitropoulos K., Barmpoutis P., Grammalidis N. (2014). Spatio-temporal flame modeling and dynamic texture analysis for automatic video-based fire detection. IEEE Trans. Circuits Syst. Video Technol..

[125] Qiu T., Yan Y., Lu G. (2011). An autoadaptive edge-detection algorithm for flame and fire image processing. IEEE Trans. Instrum. Meas..

[126] Chi R., Lu Z., Ji Q. (2017). Real-Time Multi-Feature Based Fire Flame Detection in Video. IET Image Process..

[127] Shen D., Chen X., Nguyen M., Yan W.Q. Flame Detection Using Deep Learning. Proceedings of the 2018 4th International Conference on Control, Automation and Robotics (ICCAR).

[128] Perera I.E., Litton C.D. (2011). A detailed study of the properties of smoke particles produced from both flaming and non-flaming combustion of common mine combustibles. Fire Saf. Sci..

[129] Drysdale D.D. (2016). Thermochemistry. SFPE Handbook of Fire Protection Engineering.

[130] Vojtisek-Lom M. (2011). Total diesel exhaust particulate length measurements using a modified household smoke alarm ionization chamber. J. Air Waste Manag. Assoc..

[131] Brunner C., Peynot T., Vidal-Calleja T. Combining Multiple Sensor Modalities for a Localisation Robust to Smoke. Proceedings of the 2011 IEEE/RSJ International Conference on Intelligent Robots and Systems.

[132] Morgan A. (2000). Left Luggage’-Automatic Fire Detection and the New Century. Fire Eng. J..

[133] Milke J.A., Hulcher M.E., Worrell C.L., Gottuk D.T., Williams F.W. (2003). Investigation of multi-sensor algorithms for fire detection. Fire Technol..

[134] Qualey J.R. (2000). Fire test comparisons of smoke detector response times. Fire Technol..

[135] Conforti F. (1999). Multi-sensor, multi-criteria detectors are better. Proc. AUBE.

[136] Gottuk D.T., Peatross M.J., Roby R.J., Beyler C.L. (2002). Advanced fire detection using multi-signature alarm algorithms. Fire Saf. J..

[137] Jeong J.-Y., Ryou H.-S. (2002). A study on smoke movement in room fires with various pool fire location. KSME Int. J..

[138] Liu B., Alvarez-Ossa D., Kherani N.P., Zukotynski S., Chen K.P. (2007). Gamma-free smoke and particle detector using tritiated foils. IEEE Sens. J..

[139] Bakhoum E.G. (2012). High-sensitivity miniature smoke detector. IEEE Sens. J..

[140] Aspey R.A., Brazier K.J., Spencer J.W. (2005). Multiwavelength sensing of smoke using a polychromatic LED: Mie extinction characterization using HLS analysis. IEEE Sens. J..

[141] Li M., Xu W., Xu K., Fan J., Hou D. (2013). Review of fire detection technologies based on video image. J. Theor. Appl. Inf. Technol..

[142] Çelik T., Özkaramanlı H., Demirel H. Fire and Smoke Detection without Sensors: Image Processing Based Approach. Proceedings of the 2007 15th European Signal Processing Conference.

[143] Gubbi J., Marusic S., Palaniswami M. (2009). Smoke detection in video using wavelets and support vector machines. Fire Saf. J..

[144] Ko B.C., Cheong K.-H., Nam J.-Y. (2009). Fire detection based on vision sensor and support vector machines. Fire Saf. J..

[145] Ko B., Cheong K.-H., Nam J.-Y. (2010). Early fire detection algorithm based on irregular patterns of flames and hierarchical Bayesian Networks. Fire Saf. J..

[146] Yuan F. (2011). Video-based smoke detection with histogram sequence of LBP and LBPV pyramids. Fire Saf. J..

[147] Qureshi W.S., Ekpanyapong M., Dailey M.N., Rinsurongkawong S., Malenichev A., Krasotkina O. (2016). QuickBlaze: Early fire detection using a combined video processing approach. Fire Technol..

[148] Yuan F., Fang Z., Wu S., Yang Y., Fang Y. (2015). Real-time image smoke detection using staircase searching-based dual threshold AdaBoost and dynamic analysis. IET Image Process..

[149] Li P., Zhao W. (2020). Image fire detection algorithms based on convolutional neural networks. Case Stud. Therm. Eng..

[150] Ryu J., Kwak D. (2021). Flame Detection Using Appearance-Based Pre-Processing and Convolutional Neural Network. Appl. Sci..

[151] Saponara S., Elhanashi A., Gagliardi A. (2021). Real-time video fire/smoke detection based on CNN in antifire surveillance systems. J. Real-Time Image Process..

[152] Muhammad K., Ahmad J., Baik S.W. (2018). Early fire detection using convolutional neural networks during surveillance for effective disaster management. Neurocomputing.

[153] Khan S., Muhammad K., Hussain T., Del Ser J., Cuzzolin F., Bhattacharyya S., Akhtar Z., de Albuquerque V.H.C. (2021). Deepsmoke: Deep learning model for smoke detection and segmentation in outdoor environments. Expert Syst. Appl..

[154] Valikhujaev Y., Abdusalomov A., Cho Y.I. (2020). Automatic fire and smoke detection method for surveillance systems based on dilated cnns. Atmosphere.

[155] Ruser H., Magori V. Fire Detection with a Combined Ultrasonic-Microwave Doppler Sensor. Proceedings of the 1998 IEEE Ultrasonics Symposium. Proceedings (Cat. No. 98CH36102).

[156] Schmitz H., Soltner H., Bousack H. (2010). Biomimetic infrared sensors based on photomechanic infrared receptors in pyrophilous (‘fire-loving’) insects. IEEE Sens. J..

[157] L’vov A.A., Komarov V.V., Kuzin S.A., L’vov P.A. Fire Detection and Alarm Sensor for Avionics Based on Current Loop Circuit. Proceedings of the 2018 IEEE Conference of Russian Young Researchers in Electrical and Electronic Engineering (EIConRus).

[158] Ishigaki T., Higuchi T., Watanabe K. (2007). An information fusion-based multiobjective security system with a multiple-input/single-output sensor. IEEE Sens. J..

[159] Hu H.B., Duan J.J., Lu W.J. Design of Fire Detection System Based on Digital Microholography. Proceedings of the Second Target Recognition and Artificial Intelligence Summit Forum.

[160] Zhang X., Hu J., Yang Q., Yang H., Yang H., Li Q., Li X., Hu C., Xi Y., Wang Z.L. (2021). Harvesting Multidirectional Breeze Energy and Self-Powered Intelligent Fire Detection Systems Based on Triboelectric Nanogenerator and Fluid-Dynamic Modeling. Adv. Funct. Mater..

[161] Bianchi G. (2014). Radiometer aids: Fire detection. Microw. RF.

[162] Dvorak P., Mazanek M., Zvanovec S. (2015). Fire emissivity detection by a microwave radiometer. IEEE Geosci. Remote Sens. Lett..

[163] Liu B., Han T., Zhang C. (2015). Error correction method for passive and wireless resonant SAW temperature sensor. IEEE Sens. J..

[164] Beisner E., Wiggins N.D., Yue K.-B., Rosales M., Penny J., Lockridge J., Page R., Smith A., Guerrero L. (2015). Acoustic flame suppression mechanics in a microgravity environment. Microgravity Sci. Technol..

[165] Salauddin S., Nalajala P., Godavarth B. Sound Fire Extinguishers in Space Stations. Proceedings of the 2016 International Conference on Electrical, Electronics, and Optimization Techniques (ICEEOT).

[166] Park J.H., Lee S., Yun S., Kim H., Kim W.-T. (2019). Dependable fire detection system with multifunctional artificial intelligence framework. Sensors.

[167] Qin Y.-Y., Cao J.-T., Ji X.-F. (2021). Fire detection method based on depthwise separable convolution and yolov3. Int. J. Autom. Comput..

[168] Avazov K., Mukhiddinov M., Makhmudov F., Cho Y.I. (2021). Fire Detection Method in Smart City Environments Using a Deep-Learning-Based Approach. Electronics.

[169] Ren X., Li C., Ma X., Chen F., Wang H., Sharma A., Gaba G., Masud M. (2021). Design of multi-information fusion based intelligent electrical fire detection system for green buildings. Sustainability.

[170] Park M., Ko B.C. (2020). Two-step real-time night-time fire detection in an urban environment using Static ELASTIC-YOLOv3 and Temporal Fire-Tube. Sensors.

[171] Liu P., Yu H., Cang S., Vladareanu L. Robot-Assisted Smart Firefighting and Interdisciplinary Perspectives. Proceedings of the 2016 22nd International Conference on Automation and Computing (ICAC).

[172] Ando H., Ambe Y., Ishii A., Konyo M., Tadakuma K., Maruyama S., Tadokoro S. (2018). Aerial hose type robot by water jet for fire fighting. IEEE Robot. Autom. Lett..

[173] Liljeback P., Stavdahl O., Beitnes A. SnakeFighter-Development of a Water Hydraulic Fire Fighting Snake Robot. Proceedings of the 2006 9th International Conference on Control, Automation, Robotics and Vision.

[174] Ackerman E.G.E. New WALK-MAN Robot Is Slimmer, Quicker, Better at Quenching Your Flames—IEEE Spectrum. IEEE Spectrum. https://spectrum.ieee.org/automaton/robotics/humanoids/new-version-of-walkman-is-slimmer-quicker-better-at-quenching-your-flames.

[175] L. 60^TM^ (2019). LUF 60—LUF GmbH. https://www.luf60.at/en/extinguishing-support/fire-fighting-robot-luf-60/.

[176] Fire Fighting UGV|Parosha Cheatah GOSAFER. http://www.parosha-cheatah-gosafer.com/tasks/fire-fighting-ugv/.

[177] TAF20 Robot: Firefighting Robot. https://robot.cfp.co.ir/en/newsdetail/106.

[178] ThermiteTM|Howe & Howe Technologies. https://www.howeandhowe.com/civil/thermite.

[179] DRB Fatec. http://www.drbfatec.com/html/01_business/business_0501.php.

[180] Products Archive—Brokk Global. https://www.brokk.com/product/.

